# A dataset of customer loyalty and variation in perception of customers across demographic characteristics In healthcare sector of Nigeria

**DOI:** 10.1016/j.dib.2018.08.014

**Published:** 2018-08-11

**Authors:** Taiye Tairat Borishade, Rowland E. Worlu, Oladele Kehinde, Ayodotun Ibidunni, Olaleke Ogunnaike, Joy Dirisu, Opeyemi Ogueyungbo, Fred Peter

**Affiliations:** Covenant University, Nigeria

**Keywords:** Customer, Loyalty, Perception, Demography, Healthcare

## Abstract

The data depicted on customer loyalty in the healthcare sector was concentrated on the variation in perception across demographic characteristics on the subject matter. The data focused on selected private hospitals adjudged to be the best four in Lagos State, Nigeria. In this data article, the variables for customer loyalty were categorized into (repeat purchase, brand insistence, switching restraint and customer satisfaction) for healthcare service sector in Nigeria. The data made used of the personal profile of the respondents as the independent variables to establish a link between the aims of the study and the demographic characteristics via the quantitative method. Data were gathered from 365 respondents through the use of structured questionnaire. The Kruskal Wallis Test was carried out to investigate and identify what accounted for the variation in the customers’ perception on the subject matter. The SPSS (22) was utilized to analysed the data. This dataset is presented openly for easy accessibility for a greater critical examination.

## Specification Table

**Table:** 

**Subject area**	Marketing Management
**More Specific Subject Area:**	Customer Loyalty/Healthcare
**Type of Data**	Table
**How Data was Acquired**	Primary data via questionnaire
**Data format**	Raw, analyzed, Descriptive and statistical data
**Experimental Factors**	Survey sample comprised customers in the health care sector of Nigeria. The questionnaire which comprised data on customer loyalty were completed.
**Experimental features**	Customer loyalty is an important aspect of marketing management and it covers areas such as repeat purchase, brand insistence, switching restraint, customer satisfaction etc. The healthcare customers surveyed responded to the questionnaire.
**Data source location**	Lagos State, Nigeria
**Data Accessibility**	Data are contained within this study.

## Value of data

• These data designate the demographic data of customers in the healthcare service sector of Nigeria.
• The analysis of data in this study will be valuable to the academia, industry, policy makers, consultants/practitioners and to the society at large.
• To the industry, the analysis of these data are significant to the health care service providers as it informs on the variation in customers’ perception on customer loyalty based on the personal profile of the respondents [1], [2].
• The policy makers and regulatory bodies in health care sector could use the result of this research in the formulation and the ratification of policies. Also, the Federal Ministry of Health could use the study׳s instrument to gather data on patients’ experiences with healthcare service providers so as to provide strategic policy decisions [3], [6], [7].
• The outcomes suggest that customer loyalty vary based on the demographic characteristics of the healthcare customer [4], [5].

## 1 Data

Data presented are the outcome of a survey carried out on the variation in perception of the customer on the dimensions of customer loyalty in healthcare sector of Nigeria. The data were collected via questionnaire in the four private hospitals. A total of four hundred questionnaires were administered to the customers of the four hospitals, that is, one hundred (100) questionnaires for each of the hospital. A total of 365 copies were retrieved and found to be valid and were used in the analysis. The number of questionnaires useable represents 91.25% response rate. The high response rate was due to the continuous visit and several calls made by the researcher to the respondents and probably because of the high interest the sampled respondents had in the study. This response rate is deemed reasonably high when compared to the response rate employed by previous studies.

### 1.1 Decision rule

The analysis becomes significant when the *Asymp. sig*. is less than the *p*-value of 0.05. This implies that gender, age, educational qualification, marital status and years of patronage experience significantly correlated with repeat purchase by customers (Table 1, Table 2, Table 3, Table 4).

**Table 1:** Variation in perception of the customers on repeat purchase across demographic characteristics.

**Test statistics**^a^, ^b^								
	Gender	Age	Educational qualification	Marital status	Employment status	Class of customers	Hospitals patronized	Years of patronage experience
Chi-Square	12.351	13.149	14.299	10.710	6.009	5.080	5.267	11.102
D*f*	4	4	4	4	4	4	4	4
Asymp. Sig.	0.015	0.011	0.006	0.030	0.198	0.279	0.261	0.025

a Kruskal Wallis Test.

b Grouping variable: repeat purchase.

**Table 2:** Variation in perception of the customers on brand insistence across demographic characteristics.

**Test statistics**^a^^**,**^^b^								
	Gender	Age	Educational qualification	Marital status	Employment status	Class of customers	Hospitals patronized	Years of patronage experience
Chi-Square	3.567	8.667	10.208	7.090	8.188	4.058	1.250	2.753
D*f*	4	4	4	4	4	4	4	4
Asymp. Sig.	0.468	0.070	0.037	0.131	0.085	0.398	0.870	0.600

a Kruskal Wallis Test.

b Grouping variable: brand insistence.

**Table 3:** Variation in perception of the customers on switching restraint across demographic characteristics.

**Test statistics**^a^^**,**^^b^								
	Gender	Age	Educational qualification	Marital status	Employment status	Class of customers	Hospitals patronized	Years of patronage experience
Chi-Square	1.315	16.391	15.856	10.986	7.673	3.259	5.623	1.488
D*f*	4	4	4	4	4	4	4	4
Asymp. Sig.	0.859	0.003	0.003	0.027	0.104	0.516	0.229	0.829

a Kruskal Wallis Test.

b Grouping variable: switching restraint.

**Table 4:** Variation in perception of the customers on customer satisfaction across demographic characteristics.

**Test statistics**^a^^**,**^^b^								
	Gender	Age	Educational qualification	Marital status	Employment status	Class of customers	Hospitals patronized	Years of patronage experience
Chi-Square	6.165	11.007	1.308	4.832	5.889	7.416	5.892	4.144
D*f*	4	4	4	4	4	4	4	4
Asymp. Sig.	0.187	0.026	0.860	0.305	0.208	0.115	0.207	0.387

a Kruskal Wallis Test.

b Grouping variable: customer satisfaction.

The result revealed that the difference in the perception of the customers on brand insistence by educational qualification (*λ*^2^ = 10.208, d*f* = 4, *P* < 0.05) is statistically significant.

The result revealed that the variance in the opinion of consumers on switching restraint by age (*λ*^2^ = 16.391, d*f* = 4, *P *< 0.05), educational qualifications (*λ*^2^ = 15.856, d*f* = 4, *P* < 0.05), and marital status (*λ*^2^ = 10.986, d*f* = 4, *P* < 0.05) are statistically significant.

The result revealed that the variances in the opinion of the consumers on customer satisfaction by age (*λ*^2^ = 11.007, d*f* = 4, *P* < 0.05) is statistically significant. This implies that the differences in the perception of customer on customer satisfaction can be linked to the age differences of the consumers [5].

## 2 Experimental design, materials and methods

The data for article were assembled on the variation in perception across demographic characteristics on customer loyalty in the healthcare sector. Consumers have difference perceptions on the variables of customer loyalty according to their personal profile [7]. This article therefore concentrated on the different variables of customer loyalty. The survey questionnaire was adopted for the study. This study made use of the demographic characteristics as the independent variables and the customer loyalty as the dependent variable. The Kruskal Wallis Test was carried out to investigate and identify what accounted for the variation in the customers’ perception on the subject matter. The SPSS (22) was utilized to analysed the data.
