# Supplementary material for: A dataset of customer loyalty and variation in perception of customers across demographic characteristics In healthcare sector of Nigeria
Source: Data Brief. 2018 Aug 11;20:353–7. doi: 10.1016/j.dib.2018.08.014 (PMC6117950; doi:10.1016/j.dib.2018.08.014)
Supplement: Supplementary file 1 — Supplementary material [file mmc1.docx]

**AUTHOR DECLARATION**

The authors of this data article wish to declare there are no known conflicts of interest connected with this publication and there has been no financial support for this work that could have influenced its outcome.

We, therefore declare that the manuscript has been read and approved by all named authors and that there are no other persons who satisfied the criteria for authorship but are not listed. We further confirm that the order of authors listed in the manuscript has been approved by all of us.

We declare that we have given absolute consideration to the security of intellectual property linked with this work and that there are no barriers to publication, including the timing of publication, with respect to intellectual property. In so doing we confirm that we have followed the regulations of our institutions concerning intellectual property.

We understand that the Corresponding Author is the sole contact for the Editorial process (including Editorial Manager and direct communications with the office). He/she is responsible for communicating with the other authors about progress, submissions of revisions and final approval of proofs. We confirm that we have provided a current, correct email address which is accessible by the Corresponding Author and which has been configured to accept email from (taiye.borishade@covenantuniversity.edu.ng)

Signed by all authors as follows:

**Taiye Borishade; Covenant University**

[taiye.borishade@covenantuniversity.edu.ng](mailto:taiye.borishade@covenantuniversity.edu.ng)

**Rowland E Worlu; Covenant University**

**Oladele Kehinde; Covenant University**

**Ayodotun IBIDUNNI; Covenant University**

**Olaleke Ogunnaike; Covenant University**

**Joy Dirisu; Covenant University**

**Ogueyungbo Opeyemi; Covenant University**
